# TTR-FAP: a single-center experience in Sicily, an Italian endemic area

**DOI:** 10.1186/1750-1172-10-S1-O1

**Published:** 2015-11-02

**Authors:** Anna Mazzeo, Massimo Russo, Gianluca Di Bella, Fabio Minutoli, Claudia Stancenelli, Luca Gentile, Antonio Toscano, Giuseppe Vita

**Affiliations:** 1 UOC Neurologia e MNM - Universita' di Messina - Messina, Department of Neurosciences, University of Messina - AOU G. Martino - Messina, 98100, Messina, Italy; 2 NEMO SUD Center for Neuromuscular Disorders-, AOU G. Martino - Messina, 98100, Messina, Italy; 3 Department of Clinical and Experimental Medicine, University of Messina, AOU G. Martino - Messina, 98100, Messina, Italy; 4 Department of Biomedical Sciences and Morphological and Functional Images, University of, AOU G. Martino - Messina, 98100, Messina, Italy

## Background

Familial amyloid polyneuropathy related to transthyretin gene (TTR-FAP) is a life-threatening disease transmitted as an autosomal dominant trait. Val30Met mutation accounts for the majority of the patients with large endemic foci especially in Portugal, Sweden and Japan. However, more than one hundred other mutations have been described worldwide. A great phenotypic variability among patients with late- and early-onset has been reported.

## Objective

To present a detailed report of TTR-FAP patients diagnosed in our tertiary neuromuscular center, in a 20-year period.

## Methods

Clinical informations were gathered through the database of our center.

## Results

The study involved 76 individuals carrying a TTR-FAP mutation. Three phenotypes were identified, each corresponding to a different TTR variant, homogeneous within and heterogeneous between each other: i) Glu89Gln mutation, characterised by 5th to 6th decade onset, neuropathy as presenting symptoms, early heart dysfunction, cardiomyopathy as major cause of mortality followed by dysautonomia and cachexia; ii) Phe64Leu mutation, marked by familiarity reported in one-half of cases, late onset, severe peripheral neuropathy, moderate dysautonomia and mild cardiomyopathy, death for wasting syndrome; iii) Thr49Ala mutation, distinguished by onset in the 5th decade, autonomic disturbances as inaugural symptoms which may remain isolated for many years, moderate polyneuropathy, cachexia as major cause of mortality followed by cardiomyopathy.

## Conclusions

This survey highlighted a prevalence of 8.8/1,000,000 in Sicily Island. Good knowledge of the natural history of the disease according to different TTR mutations allow clinicians to optimise multiprofessional care for patients and to offer carriers a personalized follow-up to reveal first signs of the disease.

